# Alginate Bioconjugate and Graphene Oxide in Multifunctional Hydrogels for Versatile Biomedical Applications

**DOI:** 10.3390/molecules26051355

**Published:** 2021-03-03

**Authors:** Giuseppe Cirillo, Elvira Pantuso, Manuela Curcio, Orazio Vittorio, Antonella Leggio, Francesca Iemma, Giovanni De Filpo, Fiore Pasquale Nicoletta

**Affiliations:** 1 Department of Pharmacy, Health and Nutritional Sciences, University of Calabria, 87036 Rende (CS), Italy; manuela.curcio@unical.it (M.C.); antonella.leggio@unical.it (A.L.); francesca.iemma@unical.it (F.I.); fiore.nicoletta@unical.it (F.P.N.); 2 National Research Council of Italy (CNR)—Institute on Membrane Technology (ITM), 87036 Rende (CS), Italy; e.pantuso@itm.cnr.it; 3 Children’s Cancer Institute, Lowy Cancer Research Centre, UNSW Sydney, Sydney, NSW 2031, Australia; OVittorio@ccia.unsw.edu.au; 4 School of Women’s and Children’s Health, Faculty of Medicine, UNSW Sydney, Sydney, NSW 2052, Australia; 5 ARC Centre of Excellence for Convergent BioNano Science and Technology, Australian Centre for NanoMedicine, UNSW Sydney, Sydney, NSW 2052, Australia; 6 Department of Chemistry and Chemical Technologies, University of Calabria, 87036 Rende (CS), Italy; giovanni.defilpo@unical.it

**Keywords:** hybrid hydrogels, controlled drug delivery, protein crystallization, lysozyme

## Abstract

In this work, we combined electrically-conductive graphene oxide and a sodium alginate-caffeic acid conjugate, acting as a functional element, in an acrylate hydrogel network to obtain multifunctional materials designed to perform multiple tasks in biomedical research. The hybrid material was found to be well tolerated by human fibroblast lung cells (MRC-5) (viability higher than 94%) and able to modify its swelling properties upon application of an external electric field. Release experiments performed using lysozyme as the model drug, showed a pH and electro-responsive behavior, with higher release amounts and rated in physiological vs. acidic pH. Finally, the retainment of the antioxidant properties of caffeic acid upon conjugation and polymerization processes (Trolox equivalent antioxidant capacity values of 1.77 and 1.48, respectively) was used to quench the effect of hydrogen peroxide in a hydrogel-assisted lysozyme crystallization procedure.

## 1. Introduction

Hydrogels are valuable materials in biomedicine, including tissue engineering, drug delivery, and discovery, by virtue of their toughness, softness, flexibility, and elasticity [1,2]. More importantly, the significant wetting tendency of their hydrophilic and porous surfaces are interesting features in the preparation of biomaterials suitable for different applications, including the fabrication of either release devices or templates for crystallization of biologically relevant proteins [3]. The possibility of designing effective therapeutic strategies via modulating the amount and rate of release, offers the possibility to match different therapeutic needs with a single device [4,5]. In addition, the determination of complex molecular structures through X-ray diffraction (XRD) analyses is a key finding for elucidating the molecular basis of human pathologies and discovering new therapeutic targets [6,7,8].

The functional features and chemical composition of hydrogels can be finely tuned using a combination of components with different physical or chemical properties at the nanometer or molecular level [9] and these so-called hybrid hydrogels show performances superior to those of individual components [10,11]. The high biocompatibility, low-immunogenicity, biodegradability, chemical versatility, and natural abundance of polysaccharides and proteins can be coupled with significant stability, high purity, and absence of variability between batches (e.g., molecular weight) of synthetic polymers, for the formation of versatile natural/synthetic hybrid materials [12,13]. Moreover, the incorporation of inorganic components (e.g., carbon nanostructures, CNs) via covalent or non-covalent interactions [14,15] results in organic/inorganic hybrid materials with improved properties (e.g., thermal and mechanical stability) for long-term applications [16,17]. In addition, the electrical conductivity of CNs is retained, and therefore the hydrogel properties (e.g., water uptake and affinity for a loaded therapeutic agent) can be modulated by applying an external electric field [18,19].

More recently, it has been proven that functional hydrogels with antioxidant properties can be synthesized by the conjugation of active molecules (e.g., polyphenols) to polymer chains [20,21], offering interesting solutions for mitigating oxidative stress. This is a significant challenge in both drug delivery and discovery, since the possibility to reduce oxidative damages is a key property for any material designed for interactions with living tissues [22,23], as well as in X-ray crystallography, where the oxidation of the protein sample in a crystallization droplet can lead to failure of the crystallization process [24,25].

In this work, we synthesized a multifunctional hybrid hydrogel film (HACG) obtained by co-polymerization of acrylate monomers in the presence of graphene oxide (GO), and a new sodium alginate-caffeic acid conjugate (AlgCF). The performance of HACG in biomedical applications was evaluated by exploring two main issues, namely the fabrication of a smart delivery vehicle able to modulate the release of bioactive macromolecules by electric stimulation, and the obtainment of a template able to confine and concentrate macromolecules in its porous structure, thus, facilitating crystallization under oxidation conditions.

## 2. Results and Discussion

In this study, the proposed multifunctional hybrid hydrogel (i.e., HACG) is composed of an acrylate network where AlgCF conjugate and graphene oxide (GO) are inserted as functional components, conferring peculiarities suitable for applications in biomedicine.

Acrylate polymers have been extensively studied for biomedical applications, due to their high biocompatibility and chemical versatility [26,27].

Sodium alginate (Alg) is a natural polysaccharide with β-d-mannuronic acid and α-l-guluronic acid repeating units [28], which shows the typical advantages of polysaccharides for biomedical applications, together with a high degradation rate allowing applications such as cell encapsulating and wound healing devices [29,30,31], as well as a coadjuvant in protein crystallization [32,33].

Caffeic acid (CF), which belongs to the hydroxycinnamic acids with a phenylpropenoic acid structure containing a 3,4-dihydroxylated aromatic ring attached to a carboxyl group through a trans-ethylene wire, is a powerful antioxidant that can both scavenge and inhibit the generation of free radical species [34,35]. The hexagonal lattice structure of hybridized sp^2^ carbon atoms of GO is responsible for superior electrical conductivity and ability to interact with bioactive molecules of either low or high molecular weight via π–π, hydrophobic, electrostatic interactions, and hydrogen bonding [36,37]. Furthermore, due to the presence of oxygen-rich functionalities (e.g., epoxide, phenolic, hydroxyl, and carboxylic groups), GO can be covalently incorporated in hydrogel networks via condensation/polymerization methods, obtaining hybrid hydrogels for drug delivery, biosensors, and tissue scaffold applications [38,39,40].

**Figure 1:**
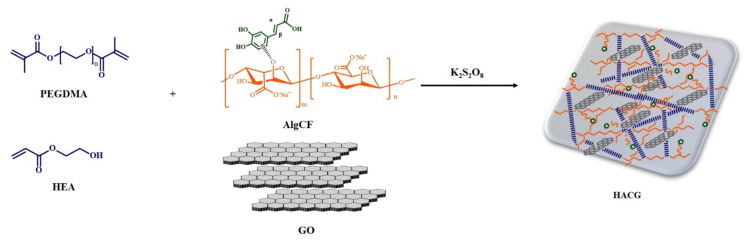
Schematic representation of the synthesis of multifunctional hybrid hydrogels.

The synthetic procedure involved the synthesis of the AlgCF conjugate, using immobilized laccase as a biocatalyst, and its subsequent co-polymerization in the presence of GO and acrylate monomer mixture, consisting of hydroxyethyl acrylate (HEA) and polyethylene glycol dimethacrylate 750 (PEGDMA) as plasticizing and crosslinking monomers, respectively (Figure 1).

### 2.1. Synthesis and Characterization of Sodium Alginate-Caffeic Acid Conjugate

AlgCF conjugate was synthesized by means of laccase chemistry via a heterogeneous catalysis approach previously developed for the conjugation of different polyphenolic compounds to polysaccharide and protein materials [41]. This methodology can be conducted in a totally green environment, ensuring the absence of any trace of toxicity and a high purity of the final product, which are key advantages for any material designed for biomedical applications. The experimental procedure involved immobilization of laccase into acrylate polymer networks. Laccase promotes a one-electron oxidation of caffeic acid (CF) [42,43] favoring the coupling by reactive groups in the Alg side chains, although the actual reaction mechanism is not well understood [44].

After purification by dialysis procedure to ensure removal of any trace of unconjugated CF, chemical characterization of AlgCF was performed by means of ^1^H-NMR and calorimetric analyses to assess the effective conjugation and the effect of CF on the thermal properties of the conjugate, respectively, while static light scattering measurements were used to estimate the average molecular weight (Mw) of Alg and AlgCF.

**Figure 2:**
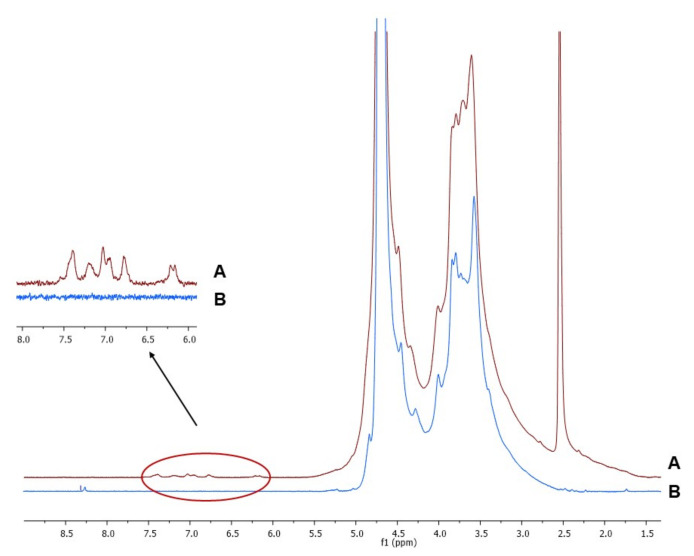
^1^H-NMR of purified (**A**) sodium alginate-caffeic acid conjugate (AlgCF) and (**B**) sodium alginate (Alg) in D_2_O.

The ^1^H-NMR spectra of Alg and AlgCF are reported in Figure 2. Signals in the range of 4.2–5.1 ppm were assigned to the Alg and AlgCF anomeric protons of β-d-mannuronic acid and α-l-guluronic acid repeating units, and to the methine protons adjacent to the carboxyl groups, the resonance of the remaining protons of sugar rings fall in between 3.2 and 4.1 ppm [45,46]. In the AlgCF spectrum, new signals not recorded in the spectrum of native Alg and assigned to CF residues were detected and considered to be experimental evidence for the effective formation of the conjugate, namely the olefinic protons at around 6.2 (α) and 7.4 (β) ppm and the aromatic protons in the range of 6.6–7.3 ppm [47].

**Figure 3:**
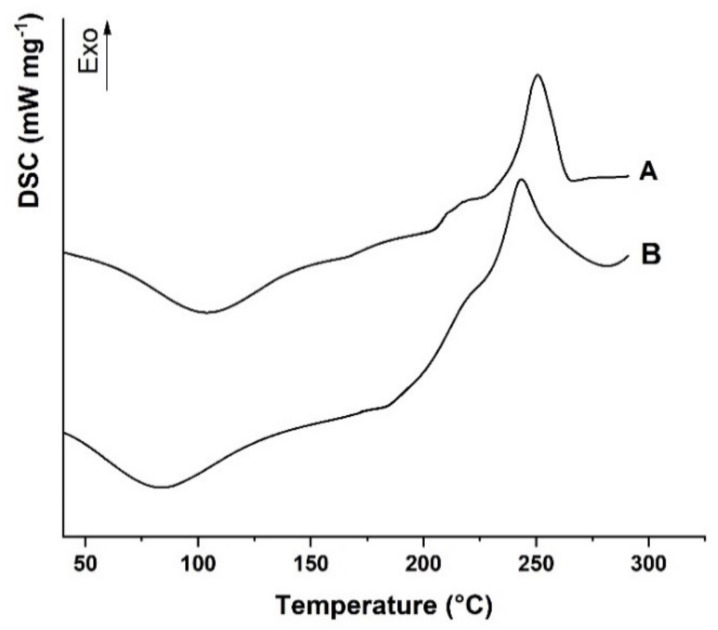
Differential scanning calorimetry (DSC) thermograms of (**A**) AlgCF and (**B**) Alg. Curves were vertically shifted for readability.

The differential scanning calorimetry (DSC) thermograms of Alg and AlgCF are shown in Figure 3.

Alg showed the typical DSC curve of polysaccharide materials with hydroxyl and carboxyl functionalities in the repeating units, with a first transition at 95 °C, assigned to the evaporation of moisture from the polymer [48], and a broad exothermic at 243 °C, expressing the formation of CO_2_, CH_4_, and H_2_O from polysaccharide chains due to decomposition at a higher temperature [49]. The decomposition involves different kinds of chemical reactions, including depolymerization, elimination of oxygen-rich functionalities in the side chains, chain scissions, recombination, and cross-linking, which can be accelerated in the presence of radical species [50].

The covalent conjugation with CF moieties was expected to enhance the thermal stability of the AlgCF conjugate, as confirmed by the shift of the exothermal degradation peak to higher temperature values (251 °C).

The results of the molecular weight determination (145 ± 15 kDa for both Alg and AlgCF) indicated that the molecular weight did not significantly change upon conjugation of CF, suggesting a functionalization degree below 10%.

### 2.2. Synthesis and Characterization of Hybrid Hydrogels

We previously reported on the possibility of covalently incorporating either carbon nanostructures (carbon nanotubes and graphene oxide) or polyphenol conjugates into acrylate hydrogels via free radical polymerization, obtaining functional materials suitable for drug delivery and healing applications [23,51].

Here, following the same synthetic approach, AlgCF and GO were simultaneously incorporated into a hydrogel network (HACG) based on HEA and PEGDMA, optimizing the reagent ratio (11.5% AlgCF, 1.15% GO, 42.30% HEA, and 45.05% PEGDMA, *w/w*) in order to maximize the amount of incorporated GO and AlgCF and the water affinity, avoiding, at the same time, hydrogel breakage upon drying. A lower amount of plasticizing and crosslinker, indeed, carried out to fragile hydrogels, while higher amounts of PEGDMA were responsible for less swellable materials.

Control samples, HAG and HAC, were synthesized by replacing AlgCF with native Alg and not inserting GO in the pre-polymerization feed, respectively, to determine the influence of either CF or GO moieties on the device performance. In detail, HAC was used to highlight the electro-responsivity in the release experiments, while HAG was useful in the crystallization experiments.

**Figure 4:**
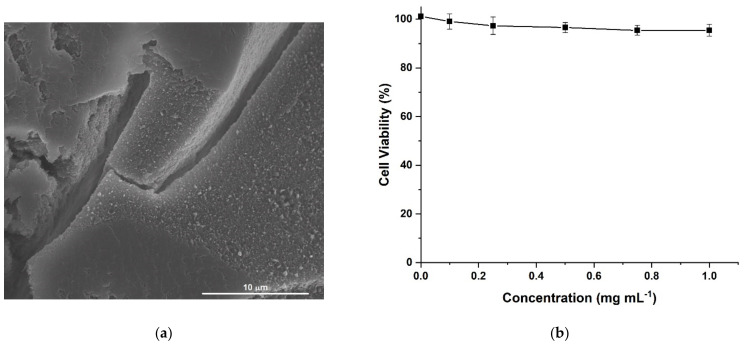
(**a**) SEM image of multifunctional hybrid hydrogel film (HACG) sample; (**b**) Human fibroblast lung cells (MRC-5) viability after 72 h incubation with pulverized HACG sample suspended in Dulbecco’s modified Eagle’s medium (DMEM) + FCS 10%.

HACG was characterized by a rough and porous surface, as per morphological investigation (Figure 4a), while human fibroblast lung cells (MRC-5) viability values higher than 94.0% ± 2.5 upon incubation of HACG (0.1 to 1.0 mg mL^−1^ concentration range, Figure 4b), confirmed its biocompatibility. These cells, indeed, are widely recognized as an in vitro model to check the toxicity of different kinds of biologically oriented materials, due to their specific metabolic features and high sensibility to almost any types of chemicals [52].

Similar results were observed when HAG and HAC were used.

Swelling experiments were performed in order to evaluate the HACG water affinity, a required key property for materials proposed for interactions with living tissues. Due to the chemical features of Alg and GO, such determination was performed in two pH conditions, mimicking an acidic (pH 5.5) and a physiological environment (pH 7.4), respectively, and in the presence or absence of electric stimulation at 12, 24, and 48 V (Table 1).

From the data in Table 1, it is evident that the carboxylic functionalities in both Alg and GO were responsible for the higher water absorption properties at pH 7.4 vs. pH 5.5, because of their different ionization statuses, carrying out electrostatic repulsion between the carboxylate anions (pKa of COOH in the range 4–5) [53]. The effect of applying an external voltage on the swelling profile can be highlighted by comparing the swelling of blank and hybrid hydrogels and introducing the swelling ratio parameter S_r_, according to Equation (1):

(1) $$S_{r}=\frac{WR_{v}-WR_{0}}{WR_{0}}\;\times \;100$$

where WR_v_ and WR_0_ are the swelling degrees at the selected voltage (12, 24, and 48 V) and 0 V, respectively.

As reported in the literature, this effect can be explained as the result of two main phenomena, i.e., ionization of the COOH functionalities and generation of an osmotic pressure between the inner and outer portions of the hydrogel network, due to the re-arrangement of mobile ions moving to the opposite electrodes [54].

The swelling degree of HAC was weakly affected by the applied voltages (Sr below 2.7 at both pH values), while the GO sp^2^ carbon layer conferred significant electro-responsivity to HACG, being more evident at acidic than physiological conditions. At pH 5.5, the larger number of available undissociated COOH could be effectively ionized by the electric stimulation, resulting in an increased electrostatic repulsion, and thus higher hydrogel swelling. On the contrary, at pH 7.4, a more significant number of COO^−^ groups was already formed even in the absence of the external voltage, and lower Sr values were recorded. Furthermore, although the ionization of COOH groups was the main driving force at 12 and 24 V, osmotic pressure started to be predominant at higher voltages (48 V), carrying out a more evident network deformation and reduced Sr values (Sr_48_ 57.8 and 20.0 vs. Sr_24_ 64.7 and 22.0 at pH 5.0 and 7.4, respectively).

### 2.3. Lysozyme Loading and In Vitro Release Studies

Electric stimulation is a valuable tool for fine tuning the delivery of therapeutic agents, in terms of both total release amount and rate, because of the possibility to modulate key parameters such as voltage intensity and duration [55]. Here, we explored the possibility of using HACG as a carrier for lysozime (LZM), a naturally occurring protein (14.4 kDa molecular weight) that shows high biocompatibility and antibacterial activities through degradation of cell walls of Gram-positive bacteria [56], and is widely proposed as a bioactive component of hydrogel systems for tissue engineering applications [57].

LZM was loaded into HAC and HACG through a soaking–drying procedure to reach the same LZM to carrier ratio in both cases (6.0%). The loading of LZM on HAC is the result of the formation of strong electrostatic interactions between the negatively charged carboxylic functionalities of AlgCF (pKa between 4 and 5) and positively charged groups on LZM (isoelectric point = 11) [58].

Release experiments were recorded as a function of pH variation, selecting the physiological (7.4) and the acidic (5.5) pH value of infection site where LZM is planned to elicit its antimicrobial activity.

For a more detailed comparison of the behavior of blank and hybrid hydrogels, experimental data were analyzed by applying four different mathematical models, considering the release kinetics as zero order (Equation (2)), first-order (Equation (3)) kinetics, or a combination of Fickian and anomalous diffusion (Equations (4) and (5)) [59].

The first model is the zero-order kinetic expressed by Equation (2) as follows:

(2) $$\frac{M_{t}}{M_{0}}=K_{0}t$$

where M_t_ is the amount of released LZM at time t, M_0_ the total amount of loaded LZM, K_0_ is the zero-order kinetic constant, and t the time of release.

Equation (3) describes a first-order kinetic as follows:

(3) $$\frac{M_{t}}{M_{0}}=a\left(1-e^{-K_{1}t}\right)$$

K_1_ is the first-order kinetic constant, t is the time of the release, and a is the release coefficient.

The third model is given by the Ritger–Peppas Equation (4):

(4) $$\frac{M_{t}}{M_{0}}=Kt^{n}$$

where K is the kinetic constant, t is the time of the release, and n is the coefficient indicating the mechanism of the release; n ≤ 0.43 indicates a Fickian diffusion mechanism, n = 0.84 a Case II transport, and 0.43 < n < 0.85 anomalous transport mechanism.

The last model is described by the Peppas–Sahlin Equation (5):

(5) $$\frac{M_{t}}{M_{0}}=K_{F}t^{1/2}+K_{a}t$$

where K_F_ and K_a_ are the kinetic constants of Fickian and anomalous diffusion, respectively.

**Figure 5:**
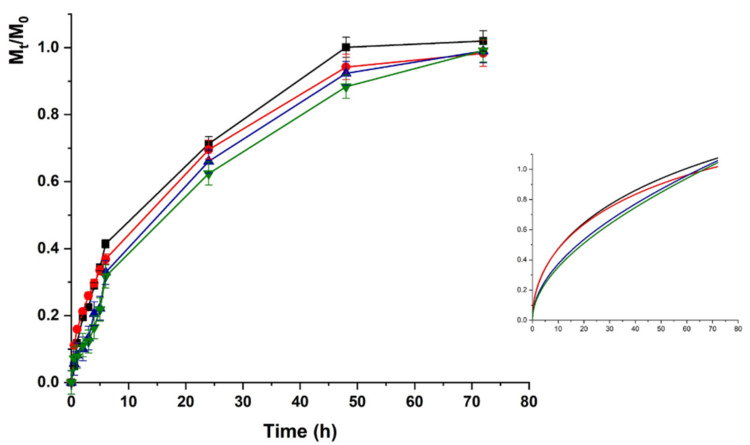
Lysozime (LZM) release profiles from HAC at pH 5.5 and (■) 0; (●) 12; (▼) 24; and (▲) 48 V. Inset: Fitting curve by Equation (5).

The LZM release profiles from HAC at acidic pH are depicted in Figure 5.

**Figure 6:**
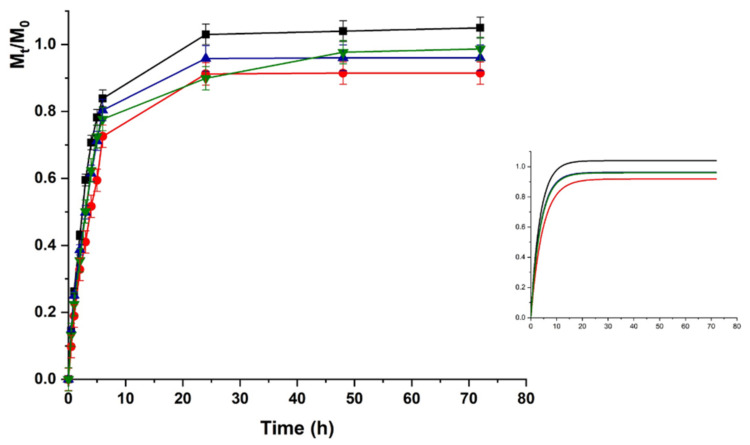
LZM release profiles from HAC at pH 7.4 and (■) 0; (●) 12; (▼) 24; and (▲) 48 V. Inset: Fitting curve by Equation (3).

Upon pH increase to 7.4 (Figure 6), the LZM release from HAC can be explained as the result of two opposite phenomena modulating the protein to hydrogel interactions. The LZM ionization equilibrium moved to the undissociated form, thus, promoting the release, while a prevalence of dissociated COOH functionalities occurred on the polymer network, enhancing the LZM affinity to the hydrogel through a higher density of negative charges. The prevalent phenomenon was the modification of ionized LZM concentration at equilibrium, resulting in an increased release at pH 7.4 vs. 5.5. The lower interactions between LZM and hydrogels at pH 7.4 were responsible for release kinetics, better described by first-order kinetics (higher R^2^ values in Table 2), while at pH 5.5 Ritger–Peppas and Peppas–Sahlin diffusion models (Equations (4) and (5)) better fitted the experimental data. Thus, the analysis of data at pH 5.5 was performed by using Equation (5).

**Figure 7:**
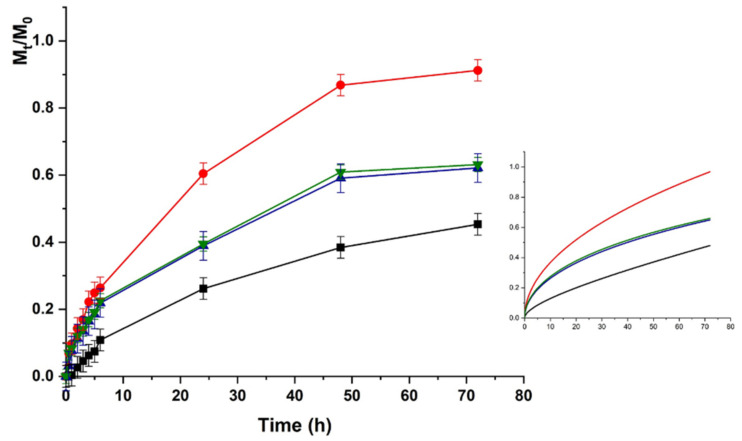
LZM release profiles from HACG at pH 5.5 and (■) 0; (●) 12; (▼) 24; and (▲) 48 V. Inset: Fitting curve by Equation (5).

**Figure 8:**
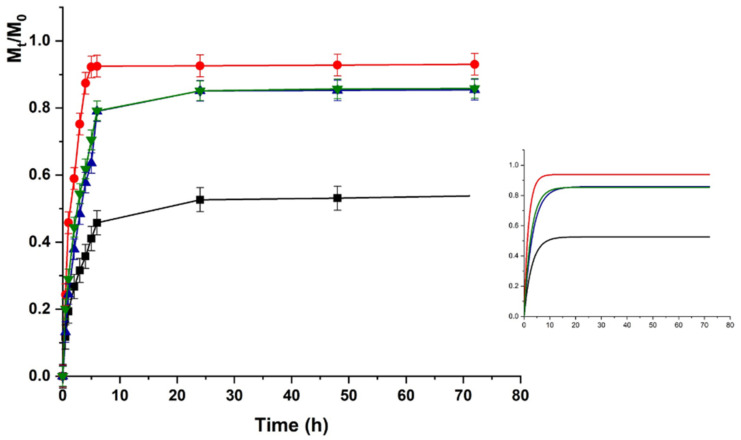
LZM release profiles from HACG at pH 7.4 and (■) 0; (●) 12; (▼) 24; and (▲) 48 V. Inset: Fitting curve by Equation (3).

The same trend was recorded for HACG, where a faster release was recorded at physiological vs. acidic pH (higher kinetic constants), although the maximum amount of release was around 50% in both cases (Figure 7 and Figure 8).

Here, the presence of GO affected the LZM to hydrogel interaction since GO is able to interact with the bioactive protein via both electrostatic (COOH) and π–π stacking interactions, thus, resulting in higher affinity at both pH conditions.

Further considerations can be done by considering the release upon application of an external electric field. As expected, no significant modification in the release amount and rate was recorded when HAC was used, due to the absence of any electro-conductive component in the polymer network. A different behavior was observed for the HACG hybrid hydrogel. The application of an electric field with different voltages resulted in a fine tuning of the delivery profiles, with the release at physiological pH being faster than that at acidic pH in all voltage conditions, due to the above-mentioned modulation of LZM to hydrogel interactions upon pH variation.

The differences in the release profiles at each selected voltage could be attributed to the modulation of both the swelling degree and the ionization state of the entire system (LZM + HACG) by the electrical stimulation. In detail, a significant increase in the release was recorded when 12 V was applied, as a consequence of the higher degree of swelling of the polymer network, promoting LZM diffusion to the surrounding environment. A further increase in the applied voltage (24 V), carried out to an enhanced ionization of COOH residues, allowed the formation of a higher number of negative charges suitable for interaction with LZM, and thus reduced the release rate/amount. A further increase in the voltage did not result in a significative modification of the release kinetics at both pHs, probably because of the formation of an equilibrium state between the LZM–HACG interaction and the strong osmotic pressure formed across the network hindering the diffusion. Further confirmations of this hypothesis can be obtained by investigating the modification of K_F_ and K_a_ kinetic constants in Equation (5) (indicating the diffusional and anomalous contributions, respectively) upon application of an external voltage. In all cases, the Fickian diffusion is the predominant effect (K_F_/K_a_ > 10), with the fast release at 12 vs. 0 V determining a significant increase in the K_F_/K_a_. When 24 and 48 V were applied, the insurgence of the osmotic stress contribution was highlighted by the simultaneous K_F_ reduction and K_a_ increase, with the K_F_/K_a_ value reduced by a half.

### 2.4. Lysozyme Crystallization upon Oxidative Stress Condition

The large wetting tendency of hydrophilic and porous surfaces of hydrogels was successfully employed in protein crystallization by virtue of their ability to reduce the activation energy to nucleation, thus, reducing the induction time and increasing the crystal growth rate [60,61]. This is importance in biomedicine, where significant research efforts have been expended for analyzing tertiary structures of proteins in order collect key information about the molecular mechanisms underlying cellular biological and pathological processes [62]. Nevertheless, chemical reactions occurring during crystallization can lead to poor reproducibility or even failure of the crystallization. Among others, the oxidization process in crystallization droplets is a key phenomenon to be considered, because of the formation of irreversible intermolecular disulfide bridges, oxidation films, and protein precipitates [24]. To address this issue, we explored the possibility of using the CF functionalities of HACG as a scavenging agent against the oxidative stress induced by H_2_O_2_ during LZM crystallization, preserving the protein tertiary structure.

To prove this hypothesis, the retainment of CF antioxidant potency upon conjugation to Alg and the further co-polymerization in the acrylate network were assessed by determining the available phenolic groups by the Folin–Ciocalteu tests [63], the Trolox (6-hydroxy-2,5,7,8-tetramethylchroman-2-carboxylic acid) equivalent antioxidant capacity (TEAC) [64], and the concentration needed for a 50% decay (IC_50_) of DPPH (1,1-diphenyl-2-picrylhydrazyl) radical [65].

The determination of available phenolic groups allowed estimating a functionalization degree of 7.7 mg CF per g of AlgCF. The TEAC value gives a clear indication about the number of radicals that can be quenched by a tested antioxidant compound; in our experimental conditions, TEAC values of 1.85 and 1.77 were obtained for free and conjugated CF (referred to the amount of conjugated CF as per Folin–Ciocalteu test), respectively, clearly demonstrating that the scavenging ability of CF was almost unchanged after insertion in the AlgCF side chains. The determination of IC_50_ values for DPPH radical (0.85 mg mL^−1^, corresponding to 6.7 µg mL^−1^ CF equivalent concentration), close to that of free CF (6.1 µg mL^−1^, *p* > 0.05) was used as a further confirmation of this statement.

HACG was found to possess an available phenolic content of 0.63 mg CF equivalent per g of dry hydrogel (suggesting an incorporation of 81.8 mg AlgCF per g), a TEAC value of 1.48 (referred to the CF content as per the Folin–Ciocalteu test), and an IC_50_ value of 27.7 mg mL^−1^ in the DPPH assay (17.45 µg mL^−1^ CF equivalent concentration).

Then, the functional hydrogel HACG was employed as substrate for LZM crystallization in standard conditions [66]. The conventional hanging drop crystallization technique was used to investigate the best conditions for obtaining good results in terms of the quality of the lysozyme crystals. In general, in a crystallization process the best results in terms of crystal quality are obtained when the crystals displayed on the drop at the end of the process are few and large. However, several parameters could influence the steps of crystal nucleation and growth. Nucleation starts when the protein solution reaches an optimal level of oversaturation. In an ideal condition, key parameters related to the solution such as pH, temperature, and precipitating agents, lead the protein solution to the narrow area of oversaturation, at which the protein can undergo a spontaneous nucleation. When the crystalline nuclei are formed, and therefore the level of over-saturation is reduced, the metastable zone is reached, where the growth of the crystal is favored [67].

As a general rule, an oversaturation condition is favored by adding suitable precipitating agents to the protein solution, such as PEG, organic solvents, or even inorganic salts such as sodium chloride. These contribute to the achievement of oversaturation in the solution by varying the chemical-physical characteristics (temperature, ionic strength, and pH). In detail, inorganic salts serve this purpose by influencing the ionic strength of the protein solution. 

In the case of the crystallization process performed in vapor phase, the polymeric hydrogel film, used as support by virtue of its porosity, promotes the establishment of a balance between the protein drop and the stripping solution present in the well, through the solvent evaporation from the drop to the stripping solution. Under these conditions, the oversaturation needed for nucleation is reached more easily. The best conditions, among those analyzed in this study, were a lysozyme protein solution concentration of 10 mg mL^−1^ in sodium acetate buffer 0.1 M, pH 4.6, and 7% *w/v* precipitating agent (NaCl) diluted in the same buffer acetate (both in the drop and in the well).

**Figure 9:**
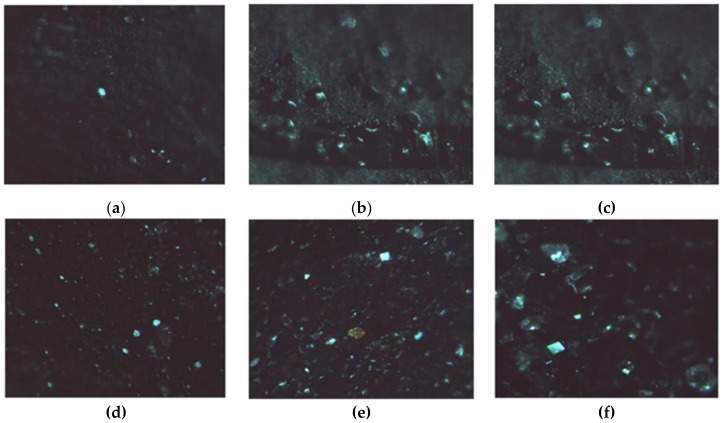
LZM crystals. (**a**–**c**) Observed on HAG; (**d**–**f**) Observed on HACG, after (**a**,**d**) 24, (**b**,**e**) 48, and (**c**,**f**) 72 h.

The formation of LZM crystals on the surface of either HAG or HACG is shown in Figure 9, confirming that the CF residues did not interfere with the process.

**Figure 10:**
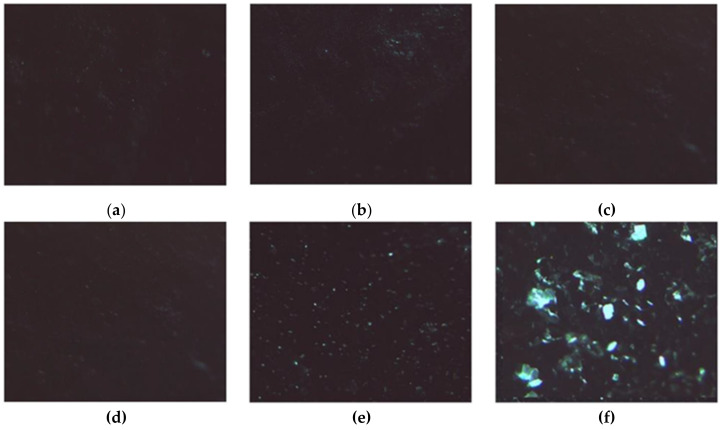
LZM crystals. (**a**–**c**) Observed on HAG; (**d**–**f**) Observed on HACG, after (**a**,**d**) 24, (**b**,**e**) 48, and (**c**,**f**) 72 h in the presence of H_2_O_2_.

In the presence of H_2_O_2_, the induced stress caused the crystallization process to fail when HAG was used as a support, obtaining small powder structures (Figure 10a–c).

Interestingly, when HACG was used as a support, the radical scavenging ability of CF residues counteracted the oxidizing activity of H_2_O_2_, resulting in the formation of well-defined LZM crystals (Figure 10d–f).

Our preliminary results can be considered to be proof of the potential applicability of HACG as functional material for LZM crystallization, with further experiments aimed to evaluate the applicability for crystallization of more complex oxidizable proteins.

## 3. Materials and Methods

### 3.1. Synthesis of Alginate-Caffeic Acid Conjugate 

AlgCF was synthesized by means of heterogeneous catalysis involving the use of a previously developed immobilized laccase as solid biocatalyst [43]. Briefly, 100 mg Alg and 10 mg CF were dissolved in 3.5 mL H_2_O containing 5% DMSO, and, after the addition of 250 mg biocatalyst (11.5 U), reacted at 37 °C under 70 rpm. After 12 h, the conjugate was purified by dialysis in 6–27/32″ dialysis tubes, MWCO 12,000–14,000 Da (Medicell International LTD, Liverpool, UK) against DMSO/H_2_O mixture solution (5%) until complete removal of unreacted CF in the washing media. Then, DMSO was removed by dialysis against water, and the solution was dried with a freeze drier (Micro Modulyo, Edwards Lifesciences, Irvine, CA, USA) to afford a vaporous solid. The presence of CF in the washing media was analyzed by high-pressure liquid chromatography (HPLC) in the following conditions: Jasco PU-2089 Plus liquid chromatography equipped with a Rheodyne 7725i injector (20 µL loop), a Tracer Excel 120 ODS-A column particle size 5 µm, 15 × 0.4 cm (Barcelona, Spain), a mobile phase consisting of acetonitrile-water containing 0.1% phosphoric acid (70:30) running at a flow rate of 1.5 mL min^−1^, a Jasco UV-2075 HPLC detector operating at 330 nm, and a Jasco-Borwin integrator (Jasco Europe S.R.L., Milan, Italy) [68].

All chemicals were from Merck/Sigma Aldrich, Darmstadt, Germany.

### 3.2. Synthesis of Hybrid Hydrogel

Hybrid hydrogels were prepared via a previously developed polymerization procedure [51] consisting of preliminary dispersion of 5.0 mg GO in 3.5 mL water containing 50 mg AlgCF water solution by a cup-horn high intensity ultrasonic homogenizer (SONOPULS) with a cylindrical tip (amplitude 70%, time 30 min). Then, 184 mg HEA and 196 mg PEGDMA were added, and the solution was purged with gaseous nitrogen for 20 min. After adding ammonium persulfate (10% *w/w*), the polymerization mixture was allowed to react at 40 °C after placement between two 5.0 × 5.0 cm^2^ glass plates, separated with a Teflon spacer (0.6 mm) and brought together by binder clips. The obtained hybrid hydrogels were extensively washed with water to remove unreacted species, and then dried overnight under vacuum at 40 °C. Control hydrogels were prepared with the same procedure without the insertion of GO (HAC) or replacing AlgCF conjugate with native Alg (HAG).

All chemicals were from Merck/Sigma Aldrich, Darmstadt, Germany.

### 3.3. Characterization Procedures

#### 3.3.1. Instruments

^1^H-NMR spectra (300 MHz, D_2_O) were recorded using a Bruker Avance 300 (Bruker Italy, Milan, Italy).

Calorimetric analyses were carried out using a DSC200 PC differential scanning calorimeter (Netzsch, Selb, Germany). Following a standard procedure, about 5.0 mg of dried sample was placed in an aluminum pan, and then sealed tightly by an aluminum lid. The thermal analyses were performed from 60 to 300 °C under a dry nitrogen atmosphere with a flow rate of 25 mL min^−1^ and heating rate of 5 °C min^−1^.

Freeze-dried grounded samples were deposited onto self-adhesive, conducting carbon tape (Plano GmbH, Wetzlar, Germany) and scanning electron microscope images were acquired using a NOVA NanoSEM 200 (0–30 kV) (Thermo Fisher Scientific, Hillsboro, OR, USA).

Static light scattering measurements (Zetasizer Nano ZS instrument, Malvern Panalytical, Malvern, UK) were performed on Alg and AlgCF water solutions with a concentration between 0.1 and 5.0 mg mL^−1^ in order to determine the average molecular weight [69]. All the solutions were filtered using a 0.22 μm filter (Millex^®^-GV syringe-driven filter unit, Merck/Sigma Aldrich, Darmstadt, Germany), and then placed in quartz cuvettes. The light intensity and its time autocorrelation function were measured at 173° scattering angle after 2 min of equilibration at 25 °C using automatic time settings.

The Debye plots were generated by using Debye’s light scattering Equation (6):

(6) $$\frac{KC}{R_{\theta }}=\frac{1}{M_{W}}+2B_{22}C$$

where *R*_θ_ is the excess Rayleigh ratio of the polymer in a solution with a polymer concentration C and M_W_ is the average molecular weight. K is the optical constant and is defined as reported in the following Equation (7):

(7) $$K=\frac{4\pi^{2}n^{2}}{N_{A}\lambda^{4}}{\left(\frac{dn}{dc}\right)}^{2}$$

where n is the solvent refractive index, dn/dc is the refractive index increment, λ is the wavelength of the incident light, and N_A_ is the Avogadro’s number. The average molecular weight was obtained from the inverse of the intercept of the linear Debye plot of KC/*R*_θ_ versus the polymer concentration C.

#### 3.3.2. Antioxidant Tests

The amount of total phenolic equivalents, expressed as CF equivalent per g of sample was determined using Folin–Ciocalteu assay, as reported in [63], using the calibration curve of the free antioxidant. The TEAC values were determined according to a previously reported protocol with slight modifications [64]. In separate experiments, AlgCF and swollen HACG at a CF equivalent concentration of 2.0 µg mL^−1^ were added to 2,2′-azinobis(3-ethylbenzothiazoline-6-sulphonic acid) (ABTS) solution in the 0–1.23 10^−4^ mol L^−1^ concentration range and incubated at 37 °C for 6 min in the dark. Then, the absorbance was measured at 734 nm and the following Equation (8) was used to calculate TEAC:

(8) $$TEAC=\frac{1}{1.9\;\left[CF\right]}$$

where 1.9 is the number of molecules that can be scavenged per mol trolox, [CF] is the CF equivalent concentration (mol L^−1^) in the sample. The maximal amount of ABTS scavenged by the CF at the tested concentration, C, was calculated by plotting the reduction of ABTS concentration against its initial concentration according to the following Equation (9):

(9) $$y=C\left(1-e^{-bx}\right)$$

where x and y are the initial ABTS concentration and the reduction in ABTS concentration, respectively.

Radical scavenging properties were evaluated measuring the inhibition (%) of the stable 2,2′-diphenyl-1-picrylhydrazyl radical (DPPH) radical by AlgCF (from 0.25 to 1.50 mg mL^−1^) and swollen HACG (from 15.0 to 30.0 mg mL^−1^), according to the literature protocol and the following Equation (10) [65]:

(10) $$Inhibition\left(\%\right)=\frac{A_{0}-A_{1}}{A_{0}}\times 100$$

where A_0_ and A_1_ are the absorbances of DPPH solution in the absence or presence of polymer samples, respectively.

The water affinity of HAC and HACG was investigated in PBS (pH 7.4) and sodium acetate buffer (pH 5.5) under different voltage conditions (0, 12, 24, and 48 V). Briefly, 1.0 cm^2^ specimens were cut from each sample, weighed, and immersed into the swelling medium at 37 °C. Excess water was removed after 24 h, and samples blotted with a tissue to remove surface moisture and weighed. The water content percentage (WR) was expressed by the following Equation (11):

(11) $$WR\left(\%\right)=\frac{W_{s}-W_{d}}{W_{d}}\times 100$$

where W_s_ and W_d_ are the sample weights in their swollen and dry state.

All chemicals were from Merck/Sigma Aldrich, Darmstadt, Germany.

#### 3.3.3. Cytotoxicity Studies

The cytotoxic effects of HACG were assessed on human fibroblast lung cells MRC-5 (ATCC CCL-171). Briefly, cells were cultured in Dulbecco’s modified Eagle’s medium (DMEM) supplemented with 10% FBS and 1% l-glutamate, grown as a monolayer at 37 °C in 5% CO_2_, and seeded into 96-well plates (100 mL per well) at a predetermined density (20,000 cells/well) to achieve 90% confluency by the endpoint of the assay.

For the cytotoxicity determination, HACG was pulverized, and incubated with MRC-5 cells after being suspended in DMEM + FCS 10% (0.1–1.0 mg mL^−1^ range of concentration). After 72 h of incubation, the media containing treatment was replaced with 10% AlamarBlue in fresh media. The metabolic activity was detected by spectrophotometric analysis by assessing the absorbance of AlamarBlue^®^ (difference between 570 nm and 595 nm) using a Bio Rad multiplate reader. Cell viability was determined and expressed as the percentage of viability of untreated control cells.

All chemicals were from Merck/Sigma Aldrich, Darmstadt, Germany.

### 3.4. Lysozyme Loading and In Vitro Release Studies

LZM was loaded into hybrid hydrogels at 6.0% (by weight) by soaking 50 mg of dried samples (HAC or HACG) with 3.5 mL LZM solution, in water (1.0 mg mL^−1^) for 3 days, and then samples were dried to a constant weight at reduced pressure in the presence of P_2_O_5_.

The in vitro LZM release was investigated by dissolution method with alternate shaking both in the absence and in the presence of an external electric voltage (12, 24, and 48 V). In separate experiments, specimens of ∼1 cm^2^ of loaded hydrogels were weighted and immersed in flasks containing 10 mL PBS (pH 7.4) and sodium acetate buffer (pH 5.5) solutions at 37.0 ± 0.1 °C in a water bath. At suitable time intervals, 1.0 mL release medium was withdrawn and replaced with fresh medium to ensure sink conditions during the experiment. After filtration (Iso-DiscTM Filters PTFE 25–4 25 mm × 0.45 μm, Supelco/Merck, Darmstadt, Germany), released LZM was measured by UV–Vis analysis on an Evolution 201 spectrophotometer (ThermoFisher Scientific, Hillsboro, OR, USA) operating with 1.0 cm quartz cells at 280 nm [70].

All chemicals were from Merck/Sigma Aldrich, Darmstadt, Germany.

### 3.5. Lysozyme Crystallization

The crystallization tests were performed by conventional hanging drop vapor diffusion method using the prepared thin films of hydrogel as crystallization supports. Lysozyme was dissolved in sodium acetate buffer (0.1 M, pH 4.6) at the initial concentration of 10 mg mL^−1^. The precipitant and stripping solutions were composed of sodium chloride, NaCl (7.0 wt.%), dissolved in the same buffer. A drop of protein solution (5 μL), pipetted on the surface of the hydrogel membrane and added with an equal volume of precipitant solution, was left equilibrating with 6 mL of stripping solution in the well. The final crystallization solution, after mixing the protein and precipitant solutions, was 5 mg mL^−1^.

To assure the result reproducibility, 5 replica experiments for each tested condition were carried out. Then, the crystallization system was incubated at 20 ± 0.1 °C before optical microscopy inspection of the droplets after time intervals of 24, 48, and 72 h. Protein crystals were observed under an optical microscope (Axiovert 25, Zeiss, Oberkochen, Germany) equipped with a video camera. The same experimental conditions were used when H_2_O_2_ (15% *w/v*) was added to the hanged drop and the reservoir solution.

## 4. Conclusions

We provided experimental evidence that a novel multifunctional hybrid hydrogel is an effective platform to either provide the electro-responsive release of biologically active molecules such as LZM or facilitate its crystallization under oxidative stress. This ability arises from the combination of the peculiar features of the network component; GO was responsible for the electro-conductivity and high affinity to LZM, while the AlgCF conjugate was the functional element with antioxidant properties.

The synthetic strategy consisted of two steps. First, AlgCF was synthesized by enzyme catalysis, and then inserted into the acrylate polymer network together with GO showing high biocompatibility and water affinity. The evaluation of the LSM release profile highlighted a pH- and electro-responsivity reliance, because of the variation of the ionization degree of carboxyl functionalities on AlgCF and GO, and the insurgence of an osmotic pressure within the swollen hydrogels upon application of an electric field.

Finally, the LZM crystallization experiments conducted in the presence of H_2_O_2_ proved the suitability of hybrid hydrogel to counteract protein denaturation, thus, facilitating the formation of well-defined crystals under oxidative conditions.

Overall, our results have shown the potential to perform subsequent studies for the development of further experimental protocols to evaluate the applicability of the proposed hydrogel system as a support for the delivery or the investigation of the three-dimensional (3D) structure of biologically relevant proteins.

## Figures and Tables

**Table 1:** Swelling behavior at equilibrium of blank and hybrid hydrogels in different pH and voltage conditions.

Sample	pH	Voltage	WR (%)	$S_{r}=\frac{WR_{v}-WR_{0}}{WR_{0}}\times 100$
HAC	5.5	0	332 ± 3.1	- - -
		12	334 ± 3.3	0.6
		24	337 ± 2.9	1.5
		48	341 ± 3.4	2.7
HACG	5.5	0	351 ± 2.8	- - -
		12	401 ± 3.0	14.2
		24	578 ± 3.2	64.7
		48	554 ± 3.1	57.8
HAC	7.4	0	605 ± 2.7	- - -
		12	611 ± 2.9	1.0
		24	617 ± 2.7	2.0
		48	620 ± 2.8	2.5
HACG	7.4	0	696 ± 3.1	- - -
		12	805 ± 3.0	15.7
		24	849 ± 2.7	22.0
		48	835 ± 2.8	20.0

**Table 2:** Kinetic parameters for LZM release obtained by applying the kinetic models.

Sample	pH	Voltage	Zero Order		First Order		Ritger–Peppas			Peppas–Sahlin			
			K_0_	R^2^	K_1_	R^2^	n	K	R^2^	K_F_	K_a_ (10^−2^)	$\frac{K_{F}}{K_{a}}$	R^2^
HAC	5.5	0	0.0178	0.8384	0.0780	0.9778	0.45	0.1590	0.9894	0.1633	0.43	38	0.9831
		12	0.0170	0.8301	0.0719	0.9490	0.43	0.1678	0.9906	0.1629	0.51	32	0.9949
		24	0.0167	0.8517	0.0621	0.9480	0.47	0.1358	0.9647	0.1417	0.21	67	0.9683
		48	0.0165	0.8655	0.0579	0.9434	0.49	0.1292	0.9670	0.1323	0.14	94	0.9724
HACG	5.5	0	0.0072	0.9490	0.0098	0.9640	0.67	0.0277	0.9808	0.0302	0.31	10	0.9744
		12	0.0155	0.8831	0.0458	0.9783	0.50	0.1117	0.9839	0.1175	0.04	293	0.9859
		24	0.0106	0.8664	0.0187	0.8646	0.48	0.0854	0.9854	0.0871	0.16	54	0.9902
		48	0.0108	0.8587	0.0196	0.8481	0.46	0.0924	0.9858	0.0921	0.17	54	0.9896
HAC	7.4	0	0.0202	0.5454	0.3006	0.9952	0.23	0.4349	0.7824	0.3785	3.10	12	0.9478
		12	0.0176	0.5958	0.1895	0.9783	0.27	0.3251	0.8145	0.2977	2.27	13	0.9555
		24	0.0186	0.5493	0.2495	0.9928	0.23	0.3950	0.7848	0.3456	2.82	12	0.9522
		48	0.0187	0.5702	0.2427	0.9896	0.38	0.3795	0.7999	0.3330	2.64	13	0.9378
HACG	7.4	0	0.0104	0.5154	0.3316	0.9825	0.20	0.2508	0.8266	0.2063	1.75	11	0.9474
		12	0.0186	0.3959	0.5843	0.9915	0.14	0.5839	0.5334	0.4509	4.25	10	0.8118
		24	0.0166	0.5205	0.2992	0.9901	0.22	0.3776	0.7383	0.3249	2.74	11	0.9313
		48	0.0168	0.4862	0.3653	0.9905	0.19	0.4241	0.7335	0.3502	3.06	11	0.9248

## Data Availability

Not Applicable.

## Sample Availability

Samples of the compounds are available from the authors.
